# Primary renal lymphoma in a uremic patient

**DOI:** 10.1093/omcr/omaf026

**Published:** 2025-05-28

**Authors:** Qiqi Qi, Manying Wen, Yicheng Huang, Yueming Liu, Shenghua Yao

**Affiliations:** Medical Affairs Department, Yuyao People’s Hospital, Ningbo, Zhejiang, China; Dalang hospital of Dongguan, Guangdong, PR China; Center for General Practice Medicine, Department of Infectious Diseases, Zhejiang Provincial People’s Hospital, Affiliated People’s Hospital, Hangzhou Medical College, Hangzhou, Zhejiang, China; Urology & Nephrology Center, Department of Nephrology, Zhejiang Provincial People’s Hospital, Affiliated People’s Hospital, Hangzhou Medical College, Hangzhou, Zhejiang, China; Department of Nephrology, Yuyao People's Hospital, Ningbo, Zhejiang, China

**Keywords:** primary renal lymphoma, maintenance haemodialysis, differential diagnosis, treatment

## Abstract

Primary renal lymphoma is a rare renal malignancy, and its occurrence in patients undergoing maintenance haemodialysis is even more uncommon. Herein, we report the case of a 57-year-old woman undergoing maintenance haemodialysis, presenting with gross haematuria. After performing enhanced magnetic resonance imaging, positron emission tomography-computed tomography, and a kidney biopsy, a diffuse large B-cell lymphoma was detected. She was subsequently treated with five cycles of a modified R-CHOP regimen consisting of rituximab, cyclophosphamide, doxorubicin, vincristine, and prednisone, and achieved partial remission, as evidenced by an abdominal computed tomography scan. At the 15-month follow-up, no further enlargement or metastasis of the residual tumour was evident. We emphasize that the clinical manifestations of primary renal lymphoma (PRL) resemble those of renal cell carcinoma, necessitating an imaging and biopsy-based differential diagnosis to avoid misdiagnosis. Although PRL has a highly aggressive phenotype and is associated with high mortality, early diagnosis and appropriate treatment can improve patient outcomes.

## Introduction

Lymphoma occurs more frequently in patients undergoing haemodialysis than in the general population. However, primary renal lymphoma (PRL), defined as lymphoma involving the kidney alone, without extensive nodal disease, is extremely rare, accounting for merely 0.62% of renal masses [1]. Diffuse large B-cell lymphoma (DLBCL) is the most common histopathological subtype of PRL [2, 3]. The clinical manifestations of PRL are similar to those of renal cell carcinoma (RCC) and include haematuria, abdominal pain, and sometimes, acute kidney injury. Therefore, differentiating PRL from RCC and other renal masses is crucial because of the difference in their treatment. A renal mass is primarily managed with radical or partial nephrectomy, whereas the standard treatment for PRL is systemic chemotherapy. PRL is highly aggressive and associated with poor survival, but early diagnosis and appropriate treatment may improve the outcome. Here, we report the satisfactory treatment of a patient on maintenance haemodialysis complicated by PRL.

## Case presentation

**Figure 1:**
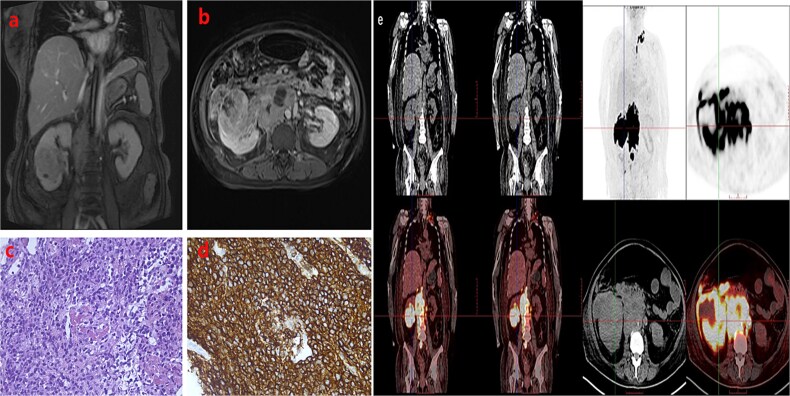
Enhanced magnetic resonance image revealing a space-occupying lesion in the right kidney. (a) Coronal section; (b) transverse section; (c) positron emission tomography-computed tomography scan revealing elevated metabolism in the right kidney, mesenteric space, and retroperitoneal celiac trunk. Histological findings of the kidney biopsy specimen: (d) haematoxylin and eosin staining (400×); (e) CD20 staining (400×): CD20 +.

**Figure 2:**
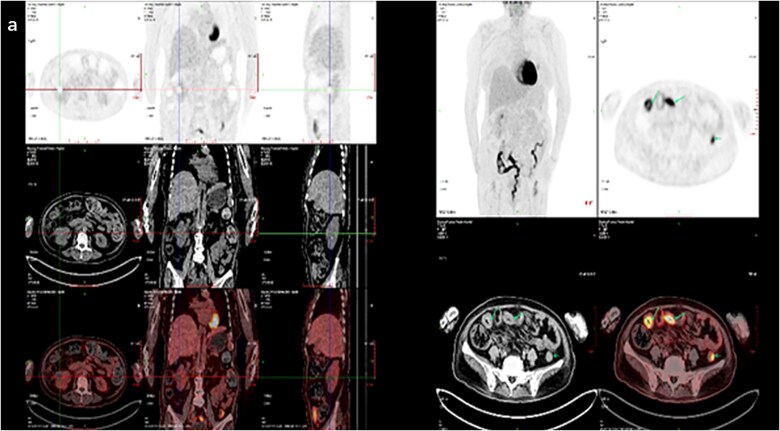
Follow-up positron emission tomography-computed tomography scan. (a) The tumour activity in the right kidney was obviously inhibited and the lesion was reduced.

A 57-year-old woman on regular haemodialysis for six months had a three-year history of diabetes mellitus, which led to end-stage kidney disease. The patient had experienced foamy urine for over 10 years but did not pay much attention to it or seek medical assistance. Three years ago, she had undergone examinations at a local hospital due to noticeable oedema. Laboratory examination revealed an albumin level of 28 g/L, urine protein of 2.7 g/24 h, creatinine of 122 μmol/l, and an estimated glomerular filtration rate of 45 ml/min. Funduscopic examination indicated diabetic retinopathy in both eyes. The patient was diagnosed with diabetic nephropathy and stage 3 chronic kidney disease. Subsequent re-examinations revealed a progressive increase in creatinine levels. Six months prior to her referral to our nephrology department, the patient had developed drug-resistant oedema accompanied by heart failure. The creatinine level had increased to 356 μmol/l, and the patient was initiated on maintenance haemodialysis. Subsequently, she also developed a bout of gross haematuria that lasted for five days and was therefore referred to our department. A physical examination revealed no abnormalities, and a routine urine test was performed. Results showed an erythrocyte count of 16 097/μl and 2+ proteinuria. A complete blood count revealed a leukocyte count of 2.79 × 10^3^/μl, a haemoglobin level of 12.7 g/dl, and a platelet count of 160 × 10^3^/μl. Enhanced magnetic resonance imaging indicated a malignant tumour in the right kidney (Fig. 1a and b). Specifically, a massive abnormal signal shadow (approximately 8.2 × 7.1 × 7.2 cm in size) with an unclear boundary was seen protruding from the outline of the kidney. The T1-weighted image displayed slightly low signal intensity, the T2-weighted image showed slightly uneven signal intensity, and the diffusion-weighted image showed high signal intensity. The apparent diffusion coefficient value was reduced, and the arterial phase of enhanced scanning revealed obvious uneven enhancement. Numerous swollen lymph nodes were evident in the right paracolic space, retroperitoneum, and around the abdominal aorta, and the lesions partially covered the inferior vena cava and abdominal aorta. ^18^F-Fluorodeoxyglucose positron emission tomography-computed tomography (PET-CT) scan revealed elevated metabolic activity in multiple masses in the abdomen, predominantly in the right kidney, mesenteric space, and retroperitoneal celiac trunk, with no lymph node involvement at other sites (Fig. 1c). To determine the nature of the renal mass, a kidney biopsy was performed. Haematoxylin and eosin staining showed large tumoral lymphoid cells with nuclear atypia and necrosis (Fig. 1d). Immunohistochemistry revealed the following phenotype: CD20 (+), CD79a (+), CD3 (−), CD5 (−), CD10 (−), CD30 (−), c-myc (+60%), Bcl-2 (+90%), Bcl-6 (+50%), Mum-1 (+50%), and Ki67 (+80%) (Fig. 1e). This confirmed the diagnosis of DLBCL. The patient was treated with a modified R-CHOP regimen—rituximab, cyclophosphamide, doxorubicin, vincristine, and prednisone—for five cycles, achieving partial remission as indicated by ^18^F-fluorodeoxyglucose PET-CT scanning (Fig. 2a).

## Discussion

A rare form of renal malignancy, PRL accounts for 0.62% of all extranodal non-Hodgkin lymphomas and approximately 0.1% of all diagnosed renal malignancies [1]. Maintenance haemodialysis complicated by this disease is even more uncommon. Primary renal lymphoma is speculated to originate from the lymph nodes of the renal sinus or the lymphatic network of the renal capsule. It typically manifests as infiltrative unilateral or diffuse bilateral renal enlargements [4, 5]. The pathogenesis of PRL remains unclear, but it may be related to perirenal lymphoid tissue invading the renal parenchyma or to chronic internal inflammation triggering malignant transformation of lymphoid tissue. Previous studies have shown that PRL may be secondary to chronic inflammatory conditions, such as Sjögren’s syndrome, chronic pyelonephritis, or other autoimmune diseases [6–8]. Primary renal lymphoma predominantly affects middle-aged and older adults, with a slightly higher incidence in men than in women. Most cases are unilateral, with the left kidney being slightly more involved than the right kidney. Although the clinical manifestations of PRL lack specificity, its imaging features do present certain characteristics. Therefore, imaging plays a crucial role in the differential diagnosis, aiding in both localisation and qualitative diagnosis. Magnetic resonance imaging provides detailed tissue characterisation of the renal vasculature. However, enhanced CT scanning is the preferred method for diagnosing PRL, owing to its simple and rapid operation and high sensitivity. PRL lacks a vascular structure; therefore, its imaging manifestations often reflect a lack of blood supply, differentiating it from more common malignancies such as clear cell renal cell carcinoma (ccRCC). There are two key points for differentiating between PRL and ccRCC by imaging. First, PRL invades the renal medulla, exhibits uneven weak enhancement, and usually lacks central necrosis. In contrast, ccRCC is a renal cortical tumour that shows an expansile growth pattern, persistent early enhancement, and peripheral distribution, with central necrosis being often present. Second, while renal blood vessel invasion and thrombus formation are rare in PRL, they are common in ccRCC [9–11]. Additionally, during CT-based diagnosis, PRL often needs to be differentiated from avascular renal tumours such as renal papillary cell carcinoma and chromophobe RCC. Moreover, when PRL is diffuse and swollen, it should also be differentiated from pyelonephritis. The main points of differentiation are as follows. First, renal papillary cell carcinoma mainly invades the renal cortex, showing expansive growth, and is prone to cystic degeneration, necrosis, and calcification, with uneven light-to-moderate enhancement on enhanced scanning. Second, chromophobe RCC originates in the renal medulla and exhibits a clear tumour boundary and, commonly, a pseudocapsule. Its density on the CT scan is higher than that of normal renal parenchyma, with calcification being common. Third, in pyelonephritis, inflammation can involve many parts of the renal parenchyma. In most patients, the lesion boundary is clear, the density of enhanced scanning is lower than that of the normal renal parenchyma, and perirenal fascia thickening and adhesion are more common.

The final diagnosis of PRL relies on a tissue biopsy and pathological examination. DLBCL is the most common histopathological subtype of PRL [2, 3]. Immunohistochemical examinations detect the the expression of all B-cell-associated markers, including CD19, CD20, CD10, Bcl-6, and CD79a. The patient in this case study exhibited a CD20 (+), CD79a (+), and Bcl-6 (+) phenotype, which fulfilled the diagnostic criteria for DLBCL. The following are the current criteria for a diagnosis of PRL [12]: (1) pathologically confirmed renal lymphoma infiltration; (2) no evidence of extrarenal lymphoma involvement of the lymph nodes or internal organs, except for the kidneys and retroperitoneal lymph nodes; (3) the absence of blood counts indicative of leukaemia or manifestations of myelosuppression; and (4) no occurrence of lymphoma at other sites within at least three months of the discovery of renal lymphoma. In the case of our patient, the PET-CT scan results revealed that the mass was localised to the right kidney, retroperitoneal mesenteric gap, and retroperitoneal abdominal trunk, with no lymph node involvement at other sites. During a 15-month follow-up period, none of the blood count results indicated leukaemia, manifestations of myelosuppression, or lymphoma development at other sites. These lines of evidence supported the diagnosis of PRL.

Although no universally accepted guidelines for the treatment of PRL currently exist, a widely agreed-upon approach is as follows. Nephrectomy is mostly recommended for the treatment of low-grade lymphomas, whereas chemotherapy without nephrectomy is considered the treatment of choice for high-grade PRL. Surgery alone often provides poor therapeutic effects and may lead to higher mortality rates [13–15]. However, nephrectomy is not recommended for patients preoperatively diagnosed with non-Hodgkin lymphoma, especially in individuals with underlying diseases such as diabetes mellitus or immunodeficiency [16, 17]. For instance, CHOP is currently the standard first-line chemotherapy regimen for DLBCL, a moderately malignant and aggressive lymphoma. The CD20 antigen is expressed in over 90% of B-cell lymphomas. Rituximab, a human-mouse chimeric anti-CD20 monoclonal antibody, induces the death of CD20-positive cells and can overcome Bcl-2-mediated resistance to chemotherapeutic agents, thereby increasing sensitivity to chemotherapy. Therefore, compared with the CHOP regimen, rituximab combined with the standard CHOP regimen (i.e. the R-CHOP regimen) significantly improves survival for patients with aggressive B-cell lymphomas and has become the new standard treatment regimen in recent years [18, 19]. Early comprehensive treatment involving surgery and the R-CHOP regimen is crucial for improving prognosis. Two patients treated with this approach have achieved tumour-free survival for more than 5 years [20]. Moreover, good treatment outcomes without surgery have been reported. A patient with left-sided PRL, who did not undergo surgery, experienced complete remission after eight cycles of treatment with the R-CHOP regimen [21]. Our patient did not undergo surgery and exhibited improvement after chemotherapy with the R-CHOP regimen, which aligns with previous findings. Regarding long-term therapeutic effects, extensive case reports comparing the therapeutic effects or clinical cure rates of radical nephrectomy + systemic chemotherapy versus systemic chemotherapy–based comprehensive treatment are lacking, warranting further research to guide clinical treatment. In general, PRL is highly aggressive and has a low survival rate, with five-year survival rates ranging from 40% to 50% [22]^.^ However, early diagnosis and appropriate treatment can improve prognosis.
